# Investigation of carbon and energy metabolic mechanism of mixotrophy in *Chromochloris zofingiensis*

**DOI:** 10.1186/s13068-021-01890-5

**Published:** 2021-02-04

**Authors:** Zhao Zhang, Dongzhe Sun, Ka-Wing Cheng, Feng Chen

**Affiliations:** 1 School of Life Sciences, Hebei University, Baoding, 071000 China; 2 Institute of Life Science and Green Development, Hebei University, Baoding, 071000 China; 3 Nutrition & Health Research Institute, China National Cereals, Oils and Foodstuffs Corporation (COFCO), Beijing, 102209 People’s Republic of China; 4 Shenzhen Key Laboratory of Marine Microbiome Engineering, Institute for Advanced Study, Shenzhen University, Shenzhen, 518060 China

**Keywords:** *Chromochloris zofingiensis*, Mixotrophy, Non-photochemical quenching, Photorespiration, Photosynthesis, RuBisCO

## Abstract

**Background:**

Mixotrophy can confer a higher growth rate than the sum of photoautotrophy and heterotrophy in many microalgal species. Thus, it has been applied to biodiesel production and wastewater utilization. However, its carbon and energy metabolic mechanism is currently poorly understood.

**Results:**

To elucidate underlying carbon and energy metabolic mechanism of mixotrophy, *Chromochloris zofingiensis* was employed in the present study. Photosynthesis and glucose metabolism were found to operate in a dynamic balance during mixotrophic cultivation, the enhancement of one led to the lowering of the other. Furthermore, compared with photoautotrophy, non-photochemical quenching and photorespiration, considered by many as energy dissipation processes, were significantly reduced under mixotrophy. Comparative transcriptome analysis suggested that the intermediates of glycolysis could directly enter the chloroplast and replace RuBisCO-fixed CO_2_ to provide carbon sources for chloroplast organic carbon metabolism under mixotrophy. Therefore, the photosynthesis rate-limiting enzyme, RuBisCO, was skipped, allowing for more efficient utilization of photoreaction-derived energy. Besides, compared with heterotrophy, photoreaction-derived ATP reduced the need for TCA-derived ATP, so the glucose decomposition was reduced, which led to higher biomass yield on glucose. Based on these results, a mixotrophic metabolic mechanism was identified.

**Conclusions:**

Our results demonstrate that the intermediates of glycolysis could directly enter the chloroplast and replace RuBisCO-fixed CO_2_ to provide carbon for photosynthesis in mixotrophy. Therefore, the photosynthesis rate-limiting enzyme, RuBisCO, was skipped in mixotrophy, which could reduce energy waste of photosynthesis while promote cell growth. This finding provides a foundation for future studies on mixotrophic biomass production and photosynthetic metabolism.

## Introduction

Microalgae are photosynthetic organisms, which began to provide the oxygen and energy needed for life 3.5 billion years ago [1, 2]. They are widely distributed and highly adaptable, and have adopted a variety of nutritional modes through natural selection including photoautotrophy, heterotrophy and mixotrophy [3–5]. Photoautotrophy and heterotrophy are two common nutrition modes which have been extensively studied. However, the underlying knowledge about mixotrophy is limited [6]. Under mixotrophic cultivation, microalgae conduct photosynthesis to fix inorganic carbon (as in photoautotrophy) while simultaneously assimilating organic carbon from the environment (as in heterotrophy). Studies have shown that, compared with photoautotrophy or heterotrophy, the growth rate, biomass accumulation, intracellular lipid content, organic carbon conversion rate and tolerance to strong light are significantly improved in certain microalgae species under mixotrophic conditions [7–9]. At present, mixotrophic cultivation has been utilized in microalgal biodiesel production [6, 10–14], resource recycling of wastewater [15], and research on eutrophic waters [16]. Therefore, further research on mixotrophy is necessary to provide important theoretical frameworks in the fields of biology, ecology, environmental governance and the production of microalgal natural products.

*Chromochloris zofingiensis* is a unicellular microalga, which could use a variety of organic carbon sources and has three nutritional modes including photoautotrophy, heterotrophy and mixotrophy [9, 17]. Furthermore, it grows fast, is easy to culture, and has a well-established genetic background [18]. These characteristics make this species an excellent model for studying mechanisms underlying mixotrophic metabolism. In addition, *C*. *zofingiensis* is rich in lipids and carotenoids, which are valuable commodities in several industries including feed additives, food production and biofuels [3]. Therefore, *C. zofingiensis* was employed in the present study. Previous study has been proposed that there is a synergistic mechanism of photosynthesis and respiration in carbon and energy metabolism in *C. zofingiensis* under mixotrophic cultivation [9]. Compared with photoautotrophy, the RuBisCO activity was declined, which indicated that the CO_2_ fixation rate is lower under mixotrophic conditions, thus conflicting with the “CO_2_ reutilization theory” [7, 9, 11]. In addition, compared to heterotrophy, the citrate synthase activity was declined, which indicated that less organic carbon entered the TCA cycle. However, the detailed underlying carbon and energy metabolic mechanism is still unknown, and two key aspects are needed to resolve:

1. Photosynthesis and glucose metabolism occur in different cellular compartments, including chloroplast, cytosol and mitochondria. How do these processes cooperate synergistically? Indeed, an enormous variety of carbon and energy metabolic processes take place in the aforementioned compartments [19]. It is interesting to note that, although chloroplasts (cyanobacteria) and mitochondria (proteobacteria) originated from different bacteria [20], each of the two organelles contains carbon and energy transporters on their membranes, which may confer them the ability to cooperate through the cytosol [21, 22]. It is reported that several transporters localized to the inner membrane of both organelles serve to interconnect energy and carbon metabolism between the stroma (chloroplast), the matrix (mitochondria) and the surrounding cytosol [22]. Key players are thought to be triose phosphate/phosphate translocators (TPTs), glucose/phosphate transporters (GPTs), ADP/ATP carriers (AACs) and other organic carbon transporters [22–24]. These transporters are well studied in higher plants but have not been studied in great detail in microalgae, and considerably less is known about how they function under mixotrophic cultivation.
2. How do photosynthesis and glucose metabolism cooperate to prevent energy loss? According to previous studies, photosynthetic light reactions can quickly absorb a large amount of light energy; however, due to the low catalytic rate and competing oxygenation activity of RuBisCO, part of the absorbed light energy cannot be used to fix CO_2_ [25]. To ensure that excess absorbed energy does not damage the photosynthetic apparatus, cells can dissipate energy in three ways: non-photochemical quenching, chlorophyll fluorescence and photorespiration [26–28]. How these three energy dissipation pathways operate under mixotrophic cultivation merits further research.

The present study was conducted to explore the detailed underlying carbon and energy metabolic mechanism of mixotrophy. The relationship between photosynthesis and glucose metabolism under mixotrophic conditions was characterized at the biochemical level at first. Then, a comparative transcriptome analysis was performed to further explore the features of carbon and energy metabolism, and a novel metabolic mechanism underlying mixotrophy was proposed. Our work established a novel mechanism for carbon and energy utilization of mixotrophy and provides a foundation for future studies on mixotrophic biomass production and photosynthetic metabolism*.*

## Results

### Interactions between photosynthesis and glucose metabolism under mixotrophic cultivation

**Fig. 1:**
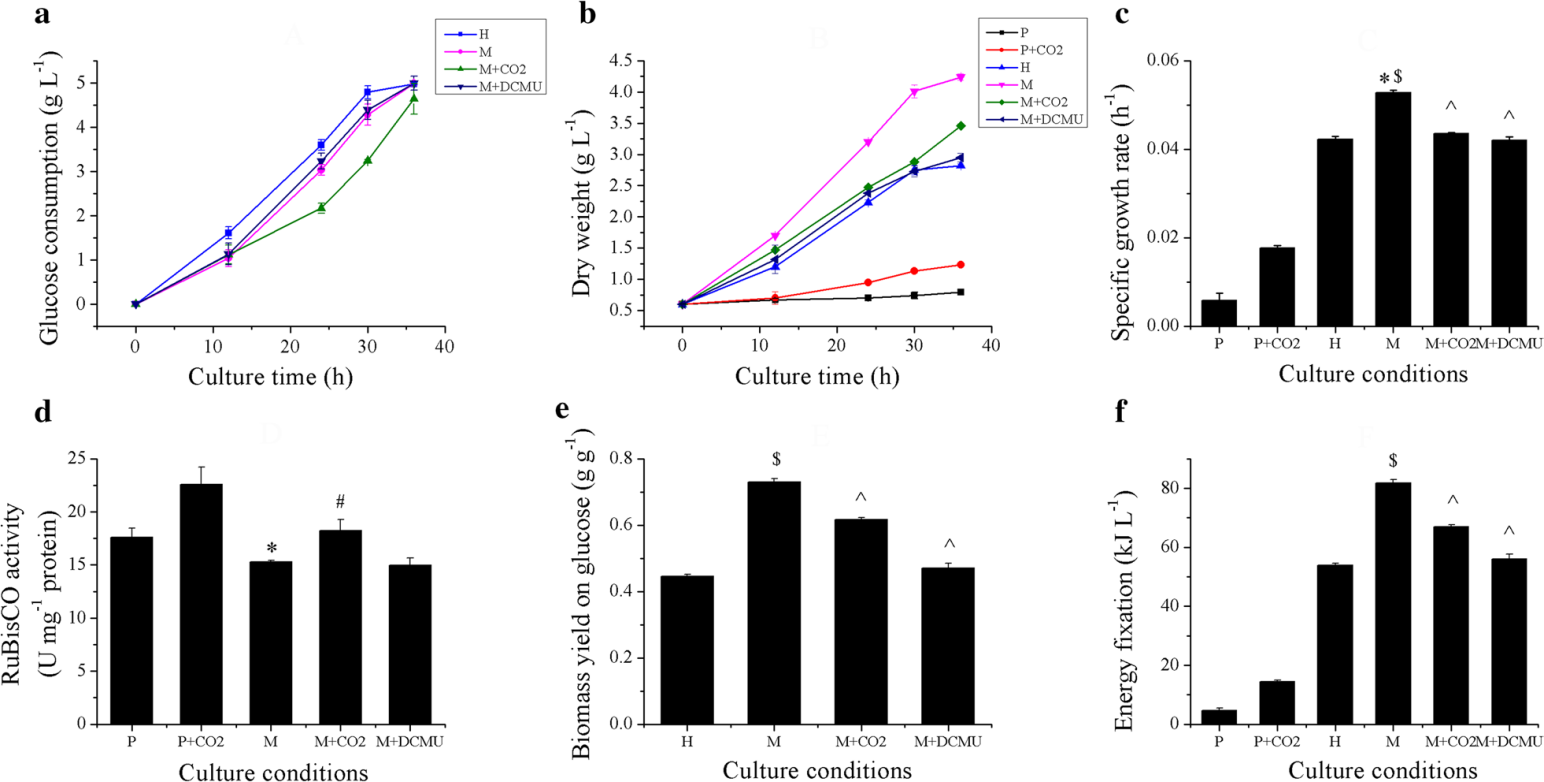
Glucose consumption, dry weight, specific growth rate (within 0–30 h), RuBisCO activity, biomass yield on glucose and energy fixation under different culture conditions. Experiments were conducted with three biological replicates. The data points were represented as values ± SD. The statistical significance of the results was tested by *t*-test (*p* < 0.05). *M significantly different with P; ^#^M + CO_2_ significantly different with P + CO_2_; ^$^M significantly different with H; ^^^M + CO_2_ and M + DCMU significantly different with M

It is well established that both photosynthesis and organic carbon assimilation provide carbon and energy for cell metabolism, and that these two biological processes can occur simultaneously under mixotrophic cultivation. Hence, a better understanding of their metabolic interactions may reveal mechanisms that contribute to fast growth rates under mixotrophy. Since carbon fixation by RuBisCO can be enhanced by increasing ambient CO_2_ concentrations [29] and 3-(3,4-dichlorophenyl)-1,1-dimethylurea (DCMU) can specifically inhibit photosynthetic electron flow from photosystem II [30]. These two factors were used to modulate the levels of photosynthesis under mixotrophic cultivation to study the effect of photosynthesis on glucose metabolism. Furthermore, a comparison between photoautotrophy and mixotrophy was also conducted to explore the effect of glucose on photosynthesis. Therefore, six culture conditions, including photoautotrophy (P), photoautotrophy with CO_2_ supplementation (P + CO_2_), heterotrophy (H), mixotrophy (M), mixotrophy with CO_2_ supplementation (M + CO_2_) and mixotrophy with 10 μM DCMU (M + DCUM), were used. Since the glucose in H, M and M + DCMU was exhausted by 36 h (Fig. 1a), all analyses were conducted before or at this time point. As shown in Fig. 1b, the maximum dry weight (DW) of M was significantly higher than the sum of H and P, which was consistent with previous research and confirmed the synergistic effect in mixotrophy [8, 9]. Therefore, it can be concluded that the additional photosynthesis could significantly increase DW. Interestingly, we observed that the DW of M increased by 1.64-fold relative to H, but glucose consumption of M was consistently lower than that of H (Fig. 1a). This indicated that although photosynthesis in M led to an increase in DW, it decreased the consumption of extracellular glucose. Besides, the specific growth rate of 0–30 h of different culture conditions were calculated according to the DW, as shown in Fig. 1c, M possessed the highest specific growth rate, 0.053 h^−1^, which is 9.13- and 1.25-fold of that in P and H, respectively. Additionally, compared with M, it was surprising to find that although CO_2_ supplementation significantly increased RuBisCO activity by 19% in M + CO_2_ (Fig. 1d), but it did not result in an increase in DW. At 36 h, we observed a DW of 2.86 g/L for M + CO_2_, which was 0.78 g/L less than for M, indicating that increased carbon fixation suppressed cell growth under mixotrophic cultivation, which also conflicts with the “CO_2_ reutilization theory” [7, 9, 11]. The glucose consumption rate of M + CO_2_ at 24 h, 30 h and 36 h was significantly lower than that of H and M, which indicated that increased CO_2_ fixation might repress glucose consumption in mixotrophy. In addition, the results of inhibition of photosynthetic linear electron transport by DCMU showed that, compared with M, with the inhibition of PSII electron transfer, DW was significantly decreased, which indicated that photosynthesis plays a crucial role in the DW increase under mixotrophy.

Two extended parameters, “biomass yield on glucose” and “energy fixation”, were introduced and calculated. The energy fixation was adopted from Zhang et al. [9], and was defined as the total energy in the increased DW. The biomass yield on glucose was defined as the ratio of DW increase to glucose [11]. As shown in Fig. 1e, f, the biomass yield on glucose and energy fixation of M was much higher than that of H, which is consistent with previous results [9]. However, it was surprising to find that CO_2_ supplementation decreased biomass yield on glucose and energy fixation. This indicated that enhanced CO_2_ fixation might result in energy and organic carbon waste under mixotrophic cultivation. Furthermore, the biomass yield on glucose and energy fixation of M + DCMU was much lower than that of M by 35.6% and 32.3%, respectively, and similar to that of H, indicating that photosynthetic light absorption is critical for glucose conversion and makes a great contribution to energy fixation. However, if the absorbed light energy is allowed to enter the photosynthetic carbon fixation reactions, it reduces glucose consumption and conversion. Therefore, a new energy and carbon metabolic mechanism likely exists under mixotrophic cultivation, which may explain the increase in glucose conversion and energy fixation.

In addition, the influence of glucose on photosynthesis under mixotrophic cultivation could also be estimated by the comparisons of P and M, P + CO_2_ and M + CO_2_. As shown in Fig. 1, since glucose could serve as both an energy and organic carbon source for cell growth, the addition of glucose significantly increased DW relative to P and P + CO_2_. However, compared with photoautotrophy, RuBisCO activity was reduced when glucose was present in the medium during mixotrophy, which of M and M + CO_2_ were found to be 13.1% and 19.3% lower than that of P and P + CO_2,_ respectively. Thus, it can be inferred that glucose might have an inhibitory effect on the photosynthetic CO_2_ fixation. Similar results have also been observed in previous research that functional enrichment analyses of comparative transcriptome sequencing showed that glucose represses photosynthetic pathways [17]. But the detailed energy and carbon metabolic mechanism of mixotrophy has not been reported yet.

To sum up, as both the photosynthesis and glucose metabolism can provide energy and organic carbon for cell metabolism, these two processes are in a dynamic balance in mixotrophy, such that enhancing one of them leads to lowering of the other.

### Sources of carbon in cells under different culture conditions

Since photosynthetic carbon fixation and glucose metabolism share common intermediates, U-13C glucose and NaHCO_3_ were applied to different culture conditions to figure out the sources of carbon in cells under different culture condition and further elucidate the interaction between the CO_2_ fixation and glucose metabolism by identifying the proportion of glucose-derived and CO_2_ fixation-derived intermediates. Five culture conditions, including heterotrophy with U-13C glucose (H + C13Glu), mixotrophy with U-13C glucose (M + C13Glu), mixotrophy with U-13C glucose and CO_2_ supplementation (M + C13Glu + CO_2_), mixotrophy with ^13^C-labeled NaHCO_3_ (M + C13NaHCO_3_ + Glu) and photoautotrophy with ^13^C-labeled NaHCO_3_ (P + C13NaHCO_3_), were examined. Eight intermediates, including glucose 6-phosphate (G6P), fructose 6-phosphate (F6P), fructose 1,6-biphosphate (FBP), dihydroxyacetone phosphate (DHAP), erythrose 4-phosphate (E4P), sedoheptulose 7-phosphate (S7P), ribulose 5-phosphate (Ru5P) and ribose 5-phosphate (R5P) were identified by HPLC–MS.

**Fig. 2:**
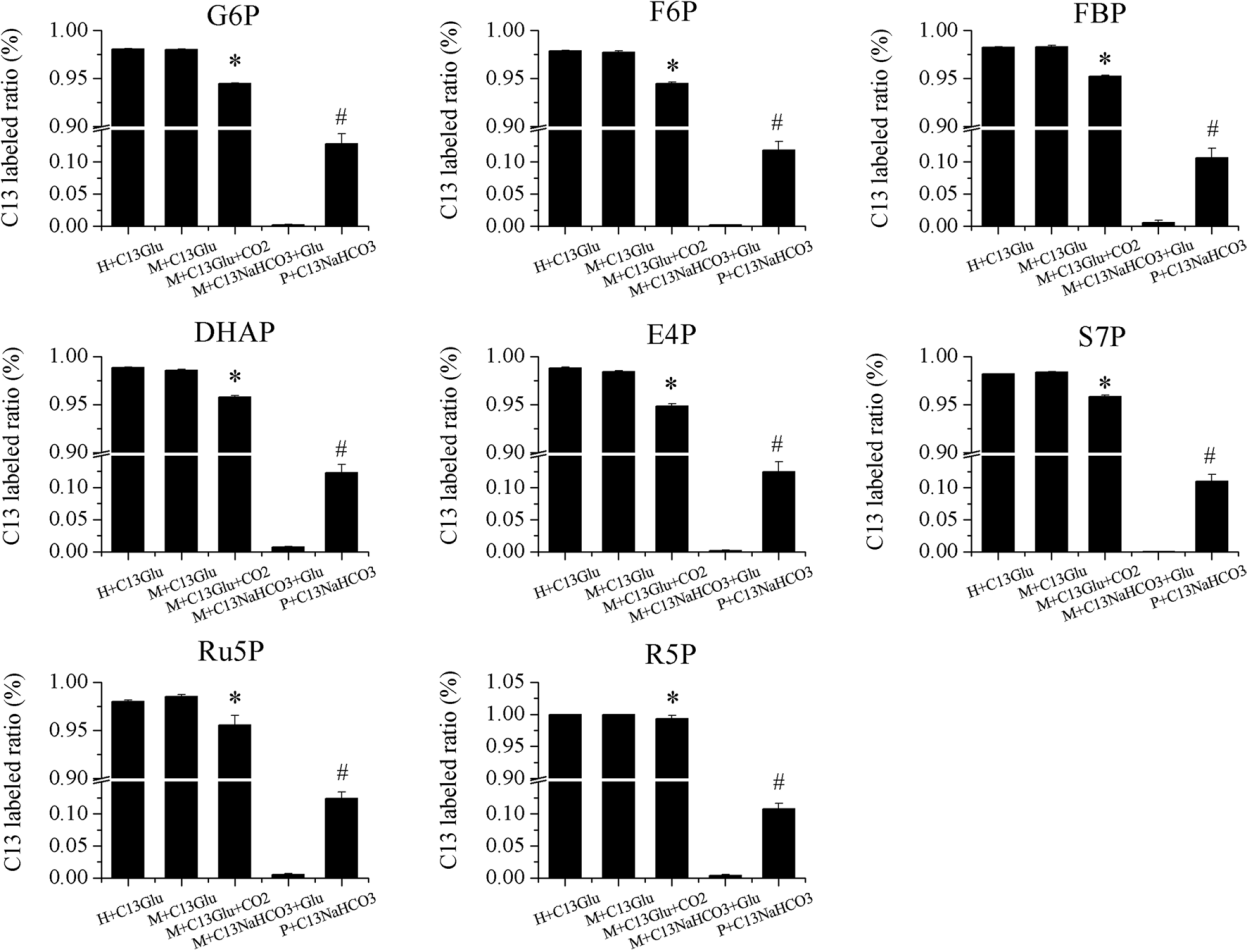
C13-labeled ratio of eight intermediates under different culture conditions. H + C13Glu: heterotrophy with C13 labeled glucose; M + C13Glu: mixotrophy with C13 labeled glucose; M + C13Glu + CO_2_: mixotrophy with C13-labeled glucose and CO_2_ aeration; M + C13NaHCO_3_ + Glu: mixotrophy with C13 labeled NaHCO_3_; P + C13NaHCO_3_: photoautotrophy with C13-labeled NaHCO_3_; G6P: glucose-6-phosphate; F6P: fructose-6-phosphate; FBP: fructose-1,6-biphosphate; DHAP: dihydroxyacetone phosphate; E4P: erythrose-4-phosphate; S7P: sedoheptulose-7-phosphate; Ru5P: ribulose-5-phosphate; R5P: ribose-5-phosphate. Experiments were conducted with three biological replicates. The data points were represented as values ± SD. The statistical significance of the results was tested by t-test (P < 0.05). *M + C13Glu + CO_2_ significantly different with M + C13Glu; ^#^P + C13NaHCO_3_ significantly differently different with M + C13NaHCO_3_ + Glu

The organic carbon labeled ratio of each intermediate was calculated and is shown in Fig. 2. All eight intermediates possessed similar variation patterns. There were no significant differences between H + C13Glu and M + C13Glu, which possessed the highest labeled ratios of around 97.5%, while the remaining 2.5% unlabeled carbon in H + C13Glu may derived from the 0.6 g L^−1^ unlabeled seeds, as its unlabeled cell components might be recycled and re-synthesized to other unlabeled metabolites. Since the organic carbon of H + C13Glu was exclusively derived from U-13C glucose, it is presumed that almost all organic carbon in M + C13Glu was also derived from glucose, which indicated that the organic carbon fixed by photosynthesis in M was limited. Moreover, compared with M + C13Glu, CO_2_ supplementation significantly decreased the labeled fraction of intermediates by about 3% of total organic carbon, from around 97.5% to 94.5% in M + C13Glu + CO_2_, which indicated that CO_2_-derived intermediates increased. As the enhancement of RuBisCO activity by CO_2_ supplementation was observed in the present study. Therefore, increases in photosynthetic CO_2_ fixation resulted in the decrease of glucose-derived intermediates. Furthermore, the labeled ratio of P + C13NaHCO_3_ was around 10%, suggesting that the unlabeled 90% might be from 0.6 g L^−1^ unlabeled seeds and atmospheric CO_2_. However, upon addition of unlabeled glucose, the labeled ratio of M + C13NaHCO_3_ + Glu was dramatically decreased to nearly zero, which indicated that glucose metabolism under mixotrophic cultivation might significantly suppress the absorption of C13NaHCO_3_. These results corresponded well with our RuBisCO activity results. Altogether, the above results established a dynamic relationship between photosynthesis and glucose metabolism under mixotrophic cultivation.

### Analysis of energy dissipation pathways

After demonstrating that photosynthesis and glucose metabolism exist in a dynamic balance under mixotrophic cultivation, we next sought to explore how these processes contribute to an increase in DW, biomass yield on glucose and energy fixation. Therefore, three major photosynthetic energy-dissipating processes (i.e., non-photochemical quenching (NPQ), chlorophyll fluorescence and photorespiration) were characterized [31].

**Fig. 3:**
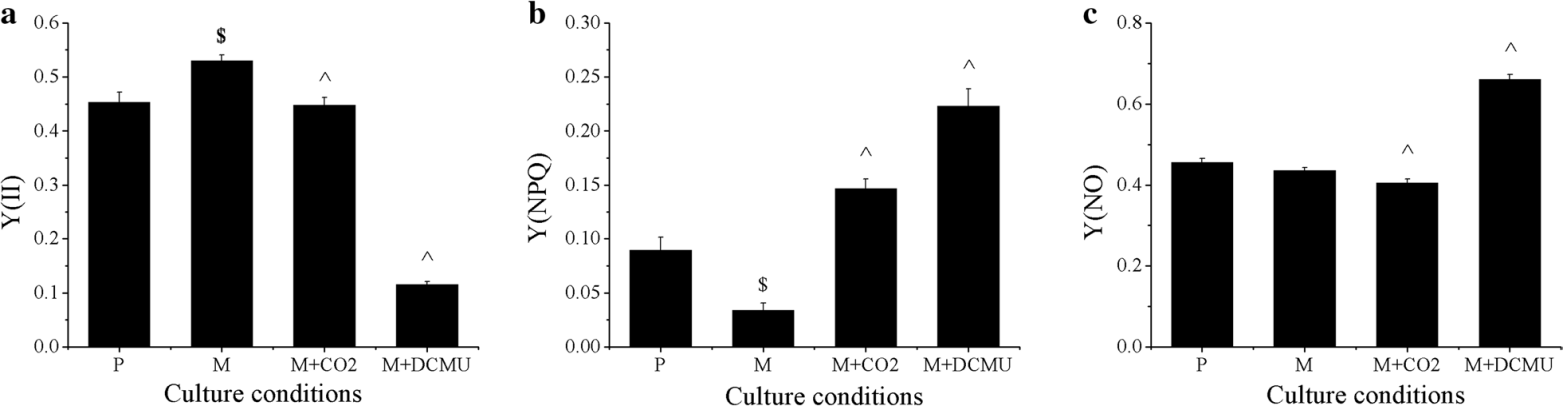
Chlorophyll fluorescence parameters under different culture conditions. Y(II) was represented as the actual quantum yield, Y(NPQ) and Y(NO) were represented as the percentage of the energy consumed by NPQ and chlorophyll fluorescence in the absorbed light energy, respectively, and Y(II) + Y(NPQ) + Y(NO) = 1. Experiments were conducted with three biological replicates. The data points were represented as values ± SD. The statistical significance of the results was tested by *t*-test (*P* < 0.05). ^$^M significantly different with P; ^^^M + CO_2_ and M + DCMU significantly differently with M

NPQ and chlorophyll fluorescence could be measured and calculated according to chlorophyll fluorescence parameters. Y(NPQ) and Y(NO) were represented as the percentage of the energy consumed by NPQ and chlorophyll fluorescence in the absorbed light energy, respectively, Y(II) was represented as the actual quantum yield, and Y(II) + Y(NPQ) + Y(NO) = 1. As shown in Fig. 3, Y(NPQ) and Y(NO) in M were 0.034 and 0.44, which represented 37.9% and 95.4% of that in P, respectively. These results suggested that mixotrophic cultivation results in less energy waste, at least under the conditions tested. Furthermore, CO_2_ supplementation in M significantly elevated the Y(NPQ) by 4.3-fold, which indicated that higher CO_2_ fixation in mixotrophy leads to higher Y(NPQ). However, different from Y(NPQ), CO_2_ supplementation slightly decreased Y(NO) from 0.44 to 0.41. Collectively, as the sum of Y(II), Y(NPQ) and Y(NO) is 1, M possessed the highest Y(II), which indicated that M has the most energy for photochemical reactions. Therefore, inhibition of linear electron transport by DCMU significantly decreased Y(II) and increased Y(NPQ) and Y(NO). These results provided an explanation for the impaired DW increase of M + DCMU that relative to M and illustrated the importance of photosynthesis for increasing DW under mixotrophic cultivation.

**Fig. 4:**
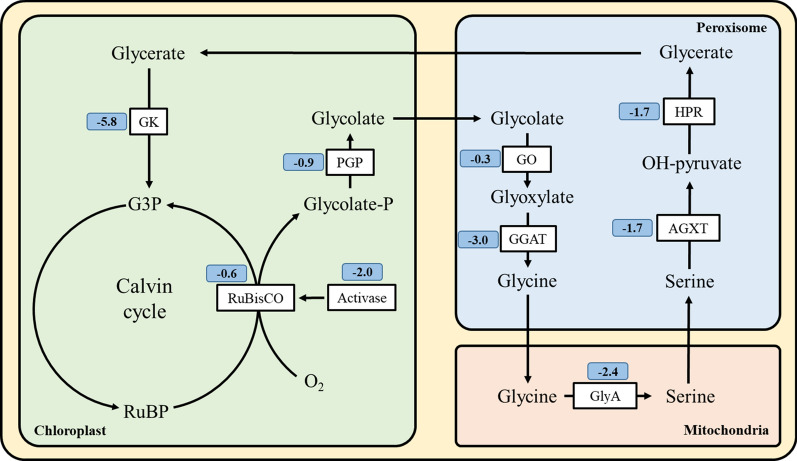
Compared with photoautotrophy, the expression changes of photorespiration biosynthesis pathway in mixotrophy. Numbers in the blue squares were expression changes that expressed in log_2_FC^a^. For genes with multiple isoforms, the isoform with the highest FPKM was selected to represent each gene. *GK* glycerate kinase, *PGP* phosphoglycolate phosphatase, *GO* glycolate oxidase, *GGAT* glutamate-glyoxylate aminotransferase, *GlyA* glycine hydroxymethyltransferase, *AGXT* serine-pyruvate transaminase, *HPR* hydroxypyruvate reductase, *G3P* glyceraldehyde 3-phosphate, *RuBP* ribulose-1,5bisphosphate

Photorespiration is another pathway that leads to energy dissipation. Therefore, we next sought to compare differences in the expression of genes involved in photorespiration between photoautotrophic cultivation and mixotrophic cultivation. Expression changes for the most highly expressed copy of each gene were shown in Fig. 4 (the trinity ID of each gene were supplied in Additional file 1: Table S1), while those of the other copies are summarized in the supplementary data. As shown in Fig. 4, expression changes were surprisingly consistent, compared with P, the most highly expressed gene copies involved in photorespiration were down-regulated in M, with expression of 7 out of the 10 being reduced by more than half. The expression of glycerate 3-kinase decreased the most, with a log_2_FC^a^ value of − 5.76. Due to the low catalytic rate and competing oxygenation activity of RuBisCO, cells need photorespiration to dissipate excess absorbed light energy [32, 33]. The observed decrease in photorespiration during mixotrophic cultivation indicated that there is an alternative route for consumption of the excess energy.

From our results, it can be concluded that cooperation between photosynthesis and glucose metabolism could significantly reduce energy loss through dissipation mechanisms (e.g., NPQ and photorespiration) associated with photosynthesis. Thus, the higher growth rate and biomass yield on glucose of M (compared with P + H) may be due to improved light energy utilization efficiency, which could also explain the decreases in NPQ and photorespiration.

### Comparative transcriptome analysis of major carbon and energy metabolic pathways

We have shown that photosynthetic carbon fixation reactions and glucose metabolism are in a dynamic balance under mixotrophic cultivation, and that faster cell growth under such conditions may in part be explained by decreases in NPQ and photorespiration. Despite this finding, the question remains about the fate of the absorbed light energy, considering the observation that Y(II) was increased under mixotrophic cultivation, whereas the levels of CO_2_ fixation and NPQ were decreased. To address this question and to better understand the detailed metabolic mechanism of mixotrophy in *C. zofingiensis*, a transcriptome analysis of photoautotrophy, heterotrophy and mixotrophy was performed, mainly focusing on changes in energy and carbon metabolic pathways and plastid membrane transporters.

1. Overall analysis of transcriptomes
  For transcriptome sequencing, we used three biological replicates from each trophic mode. A sample correlation analysis showed that the parallelism of biological replicates in each trophic mode was good and that the correlation between different trophic modes was small, which made these samples suitable for the following analysis (Additional file 2: Fig. S1).
  The numbers of differentially expressed genes (DEGs) in “photoautotrophy versus mixotrophy” (P vs. M) and “heterotrophy versus mixotrophy” (H vs. M) are summarized in Table 1. If the expression level of a gene was higher in mixotrophy, it was defined as up-regulated, otherwise, it was defined as down-regulated. Changes with |log_2_FC^a^| > 1 and *P*-value < 0.05 were defined as significant. As shown in Table 1, there were 1187 significant DEGs in P vs. M, representing 9.9% of the total genes (11,944), of which 779 genes were significantly up-regulated and 408 genes were significantly down-regulated. The number of DEGs in H vs. M was 3697, much higher than that in P vs. M, and 2025 genes were up-regulated while 1672 were down-regulated.

**Table 1:** Number of significantly different expressed genes (|log_2_FC^a^| > 1 and *P* < 0.05)

	Total	Up	Down
P vs. M	1187	779	408
H vs. M	3697	2025	1672
  As shown in Additional file 3: Fig. S2, KEGG enrichment analysis showed that only seven pathways were enriched in M vs. P, including “Proteasome,” “Ribosome,” “TCA cycle,” “Glycolysis,” “Oxidative phosphorylation,” “Carbon metabolism” and “Biosynthesis of amino acids,” which indicated that, compared with photoautotrophy, carbon and protein metabolism were significantly affected by glucose. Furthermore, 20 pathways were enriched in M vs. H. Nineteen pathways were classified in the “Metabolism” category, which were mainly distributed in photosynthesis (“Photosynthesis,” “Photosynthesis—antenna proteins,” “Carbon fixation in photosynthetic organisms,” “Carbon fixation pathways in prokaryotes,” “Porphyrin and chlorophyll metabolism”), glucose metabolism (“Pyruvate metabolism,” “Glyoxylate and dicarboxylate metabolism,” “Glycolysis/Gluconeogenesis,” “Carbon metabolism”) and fatty acid metabolism (“Fatty acid metabolism,” “Biosynthesis of unsaturated fatty acids,” “Fatty acid elongation”). The sole remaining pathway was “Ribosome,” which belonged to “Genetic information processing” and is related to protein biosynthesis. Therefore, it can be concluded from M vs. H that the additional glucose metabolism significantly altered photosynthesis and organic carbon (glucose and fatty acid) metabolism. After the general transcriptome analysis, we next sought to interrogate the mechanism underlying mixotrophic metabolism by analyzing specific carbon and energy metabolic pathways.
2. Changes in the expression of central carbon and energy metabolic pathways under mixotrophic cultivation
  The expression changes of central carbon and energy metabolic pathways, including photosynthesis, glycolysis, TCA cycle and carbon and energy exchange carriers, were summarized (the trinity ID, FPKM and log_2_FC^a^ of genes involved in the above pathways were supplied as Additional file 4. For genes with multiple copies, only the one with the highest expression was shown in Fig. 5). Since eukaryotic cells are highly compartmentalized, many energy and carbon metabolic pathways take place in different organelles or the cytosol. An elaborate network of transporters and exchange carriers establish connections between different compartments and have especially important roles for energy and carbon metabolism [21, 34–37]. Since the present work was primarily focused on carbon and energy metabolism, the expression changes of ATP and carbohydrate transporters in chloroplasts and mitochondria including chloroplast ATP/ADP carrier (CAAC), nucleotide translocator (NTT) [23, 38], triose phosphate/phosphate translocator (TPT) and glucose 6-phosphate/phosphate translocator (GPT) [23, 38] were also analyzed.

**Fig. 5:**
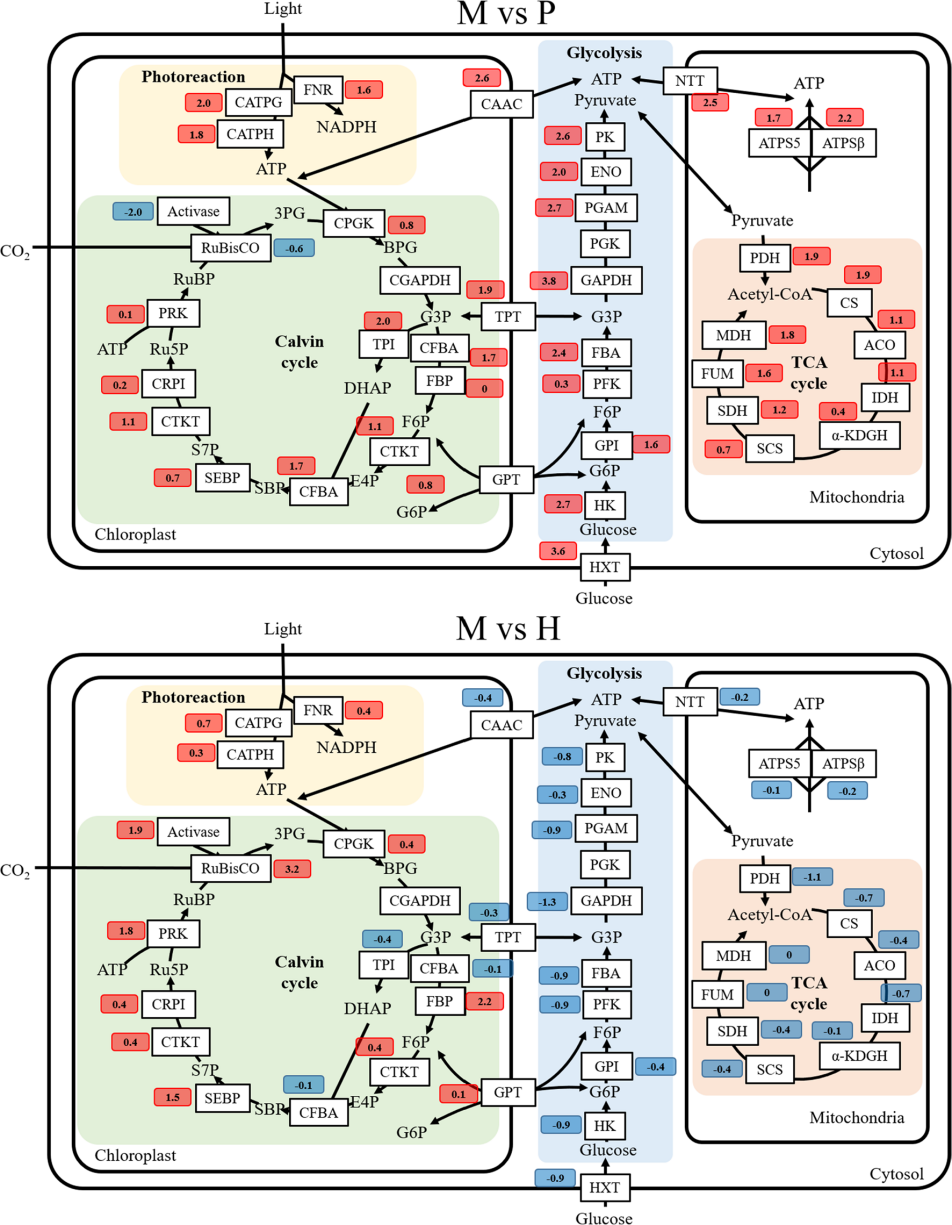
The expression changes of central carbon and energy metabolic pathways. Red square: up-regulated; blue square: down-regulated. The numbers in the colored square mean log_2_FC^a^. For genes with multiple isoforms, the isoform with the highest expression level was selected to represent each gene. Glycolytic pathway: *HXT* hexose transporter, *HK* hexokinase, *GPI* glucosephosphate isomerase, *PFK* phosphofructokinase, *FBA* fructose-1,6-bisphosphate aldolase, *GAPDH* glyceraldehyde phosphate dehydrogenase, *PGK* phosphoglycerate kinase, *PGAM* phosphoglyceromutase, *ENO* enolase, *PK* pyruvate kinase. Genes in mitochondria: *ATPS5* mitochondrial ATP synthase subunit 5, *ATPSβ* mitochondrial ATP synthase subunit β, *PDH* pyruvate dehydrogenase, *CS* citrate synthase, *ACO* aconitase, *IDH* isocitrate dehydrogenase, *α-KDGH* a-ketoglutarate dehydrogenase, *SCS* succinyl-CoA synthetase, *SDH* succinate dehydrogenase, *FUM* fumarase, *MDH* malate dehydrogenase. Genes in chloroplast: *CATPG* chloroplast ATP synthase gamma chain, *CATPH* chloroplast ATP synthase delta chain, *FNR* ferredoxin-NADP reductase, *CPGK* chloroplast phosphoglycerate kinase, *CGAPDH* chloroplast glyceraldehyde phosphate dehydrogenase, *TPI* triose phosphate isomerase, *CFBA* chloroplast fructose-1,6-bisphosphate aldolase, *FBP* fructose-1,6-bisphosphatase, *CTKT* chloroplast transketolase, *SEBP* sedoheptulose-1,7-bisphosphatase, *CRPI* chloroplast ribose 5-phosphate isomerase, *PRK* phosphoribulokinase. Carbon and energy transporters on chloroplast and mitochondria: *CAAC* chloroplast ATP/ADP carrier, *TPT* triose phosphate/phosphate translocator, *GPT* glucose 6-phosphate/phosphate translocator, *NTT* nucleotide translocator. Metabolites: *G6P* glucose-6-phosphate, *F6P* fructose-6-phosphate, *G3P* glycerate-3-phosphate, *E4P* erythrose-4-phosphate, *S7P* sedoheptulose-7-phosphate, *Xu5P* xylulose-5-phosphate, *Ru5P* ribulose-5-phosphate, *R5P* ribose-5-phosphate, *3PG* 3-phosphoglycerate, *BPG* d-glycerate 1,3-diphosphate, *SBP* sedoheptulose 1,7-bisphosphate, *RuBP* ribulose-1,5-bisphosphate

As shown in Fig. 5, compared with photoautotrophy, due to the presence of glucose, it was not surprising to find that the glycolysis pathway and TCA cycle pathway were all dramatically up-regulated in mixotrophy. Expression of over 80% of the genes increased by more than 100%. For photosynthesis, the expression of genes involved in production of ATP and NADPH was significantly increased, suggesting that the energy conversion efficiency in mixotrophy was increased, which may be related to a decrease in NPQ. However, the expression levels of photosynthetic rate-limiting enzymes (e.g., RuBisCO and RuBisCO activase) were significantly down-regulated, which is consistent with our biochemical measurements. These results indicate a decrease in CO_2_ fixation and suggest that the increase in ATP produced by photophosphorylation was not used for CO_2_ fixation, but rather for other metabolic pathways. Surprisingly, with the exception of RuBisCO, other enzymes involved in carbon fixation were significantly up-regulated. It is well established that chloroplasts are the site of many anabolic reactions including starch, lipid and amino acid biosynthesis [38, 39]. Enzymes involved in carbon fixation may also participate in these pathways. Thus, chloroplast organic carbon metabolism was elevated. Additionally, chloroplast organic carbon transporters were up-regulated under mixotrophic cultivation, especially triose phosphate translocators, whose expression levels increased by more than threefold. This suggested that compared with P, more intermediates of glycolysis could directly enter the chloroplast in M. Considering the down-regulation of RuBisCO, it was reasonable to propose that, rather than carbon derived from CO_2_ fixation by RuBisCO, the intermediates of glycolysis were used as a carbon source for organic carbon metabolism in the chloroplast under mixotrophy. In line with this, previous studies have shown that the accumulation of 3-carbon sugars in chloroplasts inhibits the Calvin cycle [37, 40]. Thus, under mixotrophic cultivation, the transport of organic carbon to chloroplasts may lead to the inhibition of CO_2_ fixation. Therefore, the main rate-limiting enzyme, RuBisCO, could be considered as being skipped (or bypassed), allowing more of the absorbed light energy to be saved and/or utilized in chloroplast organic carbon metabolism. Furthermore, we found that the chloroplast ATP transporter was also significantly up-regulated, suggesting increased transport of cytosolic ATP into chloroplasts to provide energy for organic carbon metabolism.

A comparison between heterotrophy and mixotrophy was also performed. As shown in Fig. 5, due to the dynamic balance between photosynthesis and glucose metabolism, glycolysis and the TCA cycle were moderately suppressed by photosynthesis. In terms of photosynthesis, compared with heterotrophy, the expression of RuBisCO and RuBisCO activase were significantly up-regulated under mixotrophy. However, TPI and FBA, two key enzymes involved in carbon fixation, were unexpectedly down-regulated. This may be due to a decrease in G3P transport from the cytoplasm to the chloroplast. In addition, the expression of genes involved in the production of ATP and NADPH by the photosynthetic light reactions was also significantly increased under mixotrophy. As more energy was generated by the light reactions, there was less need to transport ATP into chloroplasts. Thus, the expression of CAAC decreased. In turn, less energy was needed from mitochondria, and thus the expression level of NTT, which functions in mitochondrial ATP transport, was decreased. Lastly, as less organic carbon was used for ATP production, more organic carbon would be available to support high biomass yields on glucose under mixotrophic cultivation.

**Fig. 6:**
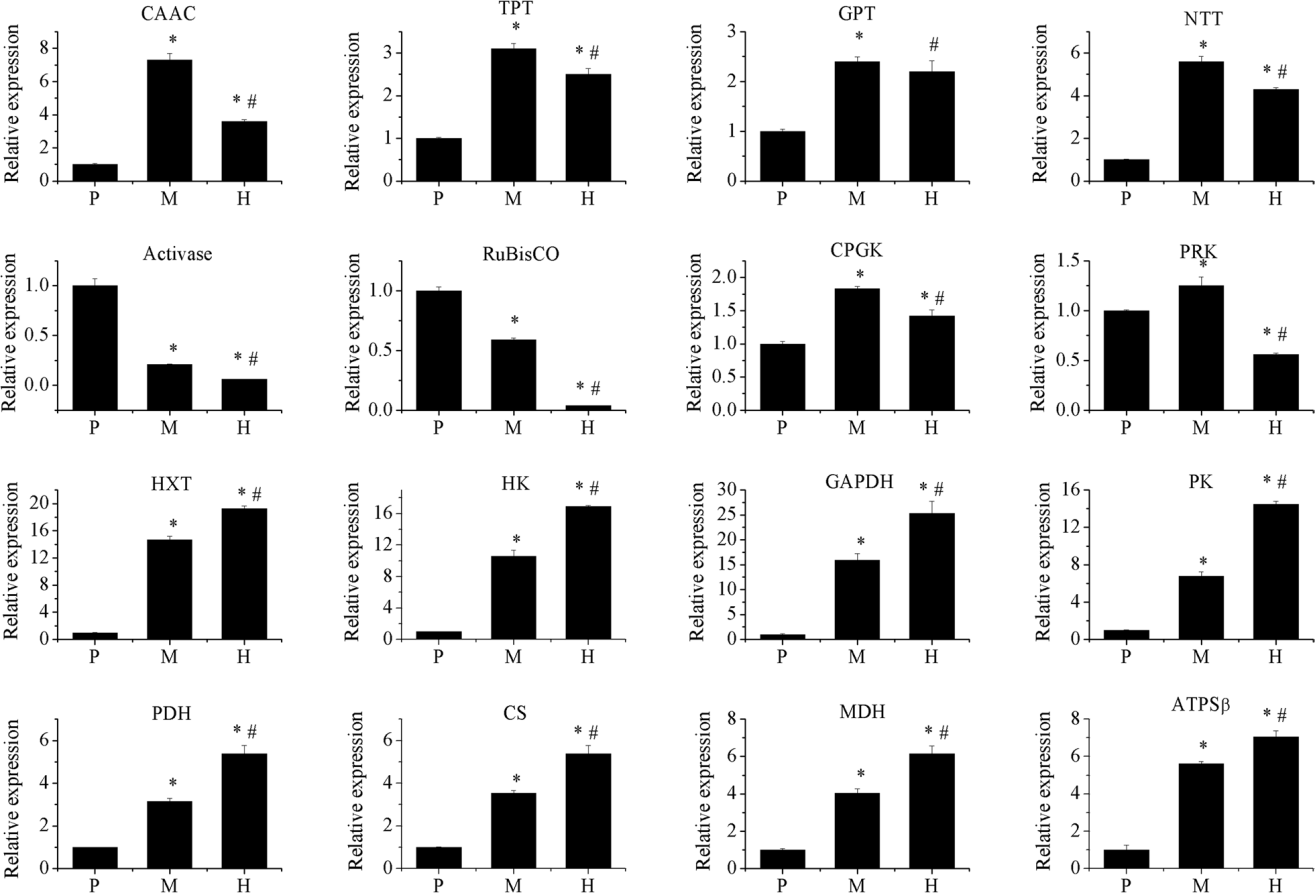
The differentially expressed genes detected by RT-qPCR. Experiments were conducted with three biological replicates. The data points were represented as values ± SD, *significantly different with P; ^#^significantly different with M. *CAAC* chloroplast ATP/ADP carrier, *TPT* triose phosphate/phosphate translocator, *GPT* glucose 6-phosphate/phosphate translocator, *NTT* nucleotide translocator, activase RuBisCO activase, *CPGK* chloroplast phosphoglycerate kinase, *PRK* phosphoribulokinase, *HXT* hexose transporter, *HK* hexokinase, *GAPDH* glyceraldehyde phosphate dehydrogenase, *PK* pyruvate kinase, *PDH* pyruvate dehydrogenase, *CS* citrate synthase, *MDH* malate dehydrogenase, *ATPSβ* mitochondrial ATP synthase subunit β

To experimentally verify the accuracy of transcriptome data, 16 genes, distributing in membrane transporters, Calvin cycle, glycolysis and TCA cycle, were selected to perform qRT-PCR. As shown in Fig. 6, all the selected genes showed the similar patterns as those identified in comparative transcriptome analysis. Therefore, the transcriptomic data were reliable.

### A novel mechanism underlying mixotrophic metabolism in *Chromochloris zofingiensis*

**Fig. 7:**
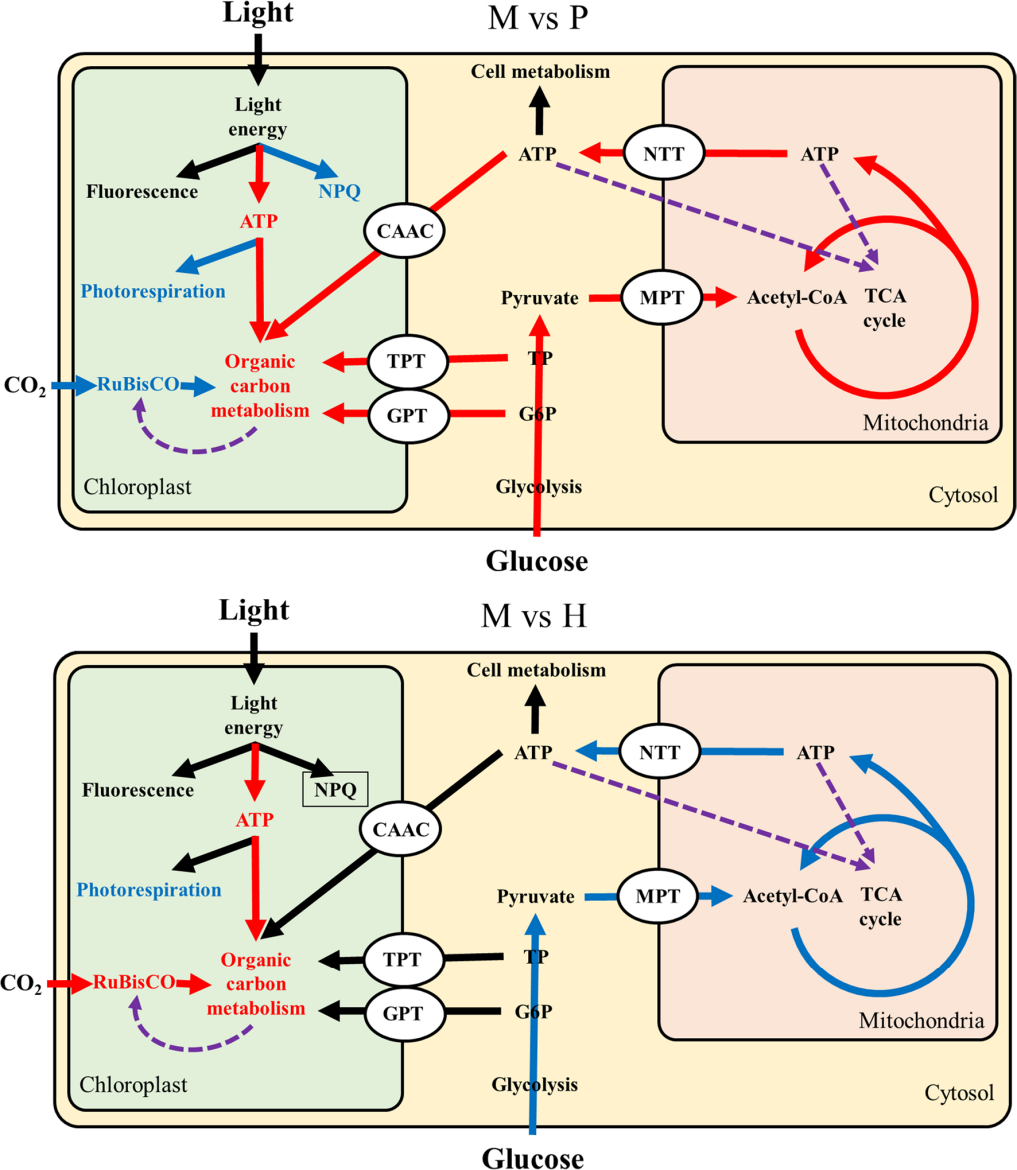
Proposed mixotrophic metabolic mechanism in *C. zofingiensis* (red arrow: up-regulated bioprocess; blue arrow: down-regulated bioprocess; black arrow: no significantly different; purple dashed arrow: feedback regulation)

Based on the above results, a mixotrophic metabolic mechanism in *C. zofingiensis* was proposed as follows: as shown in Fig. 7, compared with photoautotrophy, carbon metabolism was significantly enhanced in chloroplasts under mixotrophic cultivation. Intermediates of glycolysis as well as ATP in the cytoplasm were transported into the chloroplast for organic carbon metabolism, and the increased concentration of organic carbon in the chloroplast would feedback-suppress RuBisCO activity and gene expression. Furthermore, chloroplast organic carbon can be supplied by glycolysis, thus eliminating the demand for carbon fixation by RuBisCO. Therefore, the maximum rate-limiting step of photosynthesis (i.e., fixation of inorganic carbon by RuBisCO) was skipped, which resulted in lower levels of NPQ and photorespiration. Since ATP derived from photophosphorylation was increased, and two ATP-consuming processes, namely photorespiration and CO_2_ fixation, were down-regulated, more photosynthetically derived ATP was available for chloroplast organic carbon metabolism. In addition, compared with heterotrophy, as photosynthesis could provide ATP for organic carbon metabolism, the need for ATP transport from the cytoplasm to the chloroplast was reduced. Therefore, ATP derived from glucose metabolism would be expected to accumulate in the cytoplasm and mitochondria, which would, in turn, feedback suppress the TCA cycle. As such, there was less need for organic carbon decomposition. Considering that photosynthesis still results in the fixation of a certain amount of CO_2_ under mixotrophic cultivation, glucose uptake was decreased compared to heterotrophic cultivation conditions.

Our work represents the first proposal of a detailed mixotrophic metabolic mechanism in *C. zofingiensis*, which establishes a framework for future theoretical studies and industrial application.

## Discussion

As the connections among different cell compartments and the ways of corporation between different metabolic pathways in eukaryotic organisms could be many and varied, their carbon and energy metabolisms are complex and fascinating [29, 34, 35, 37–39]. Recent results showed that diatoms optimize their photosynthetic efficiency via elaborate interactions between plastids and mitochondria [41]. Besides, several studies have also noticed the synergistic photosynthesis and glucose metabolism in certain microalgae species under mixotrophic cultivation, but its carbon and energy metabolic mechanism is currently poorly understood [7, 9, 11]. The present study provided an interaction scenario between photosynthesis and glucose metabolism in *C. zofingiensis* under mixotrophic cultivation, in which the additional organic carbon source can replace the RuBisCO-fixed CO_2_ for the organic carbon metabolism in the chloroplast, and provides sufficient precursors for the utilization of light energy. Thus, rather than CO_2_ fixation, photosynthesis became mainly employed for light energy fixation in mixotrophy. This was similar with previous results in cyanobacteria, where the authors indicated that the photosystems are mainly employed for reducing equivalents and energy supplies with limited CO_2_ fixation during mixotrophic growth [42]. As a result, the photosynthesis rate-limiting enzyme, RuBisCO, was skipped in mixotrophy, which could reduce energy waste of photosynthesis while promote light energy utilization efficiency and cell growth. And similar results that mixotrophy can confer a higher growth rate than the sum of photoautotrophy and heterotrophy have also been reported in other microalgal species [7, 43].

Glucose and its metabolic intermediates and ATP were presented both in photosynthesis and glucose metabolism, which could also function as regulators in many biological processes, and might coordinate photosynthesis and glucose metabolism in mixotrophy. It was reported that chloroplast-derived carbohydrates could regulate cellular metabolisms [34]. For instance, triose phosphates can trigger the expression changes of cytosolic transcription factors, and organic carbons were reported to feedback regulate photosynthesis [44]. Besides, evidence have showed that post-translational regulation may affect the Calvin cycle enzymes in microalgal species [45]. Apart from the regulations of organic carbons on photosynthesis, the regulation of photosynthesis on glucose uptake has also been reported in the present study. Besides, citrate synthase, a key enzyme of TCA cycle, was reported to be regulated by the ratio of ATP/ADP [46]. As photoreaction could provide ATP for cell metabolism, it was not hard to understand the downregulation of TCA cycle in mixotrophy. In general, the mutual regulation of photosynthesis and glucose metabolism is a complex process. The mixotrophic metabolic mechanism proposed in this study is the result of their collaboration.

Previous research showed that, adg1-1/tpt-2, an *Arabidopsis thaliana* double mutant impaired in acclimation to high light with an 80–90% inhibition of ETR, could be rescued by exogenously supplied sugars (i.e., glucose and sucrose) [47, 48]. A scenario was proposed that the fed sugars would be transported into chloroplast and used for anabolism. However, a recent review pointed out that this scenario would entail the assumption that CO_2_ fixation by Calvin–Benson cycle would be minimized or even blocked through sugar feeding, which awaits to be tested experimentally [34]. The present study showed that the intermediates derived from exogenous glucose would directly enter the chloroplast and replace RuBisCO-fixed CO_2_ to provide carbon sources for chloroplast organic carbon metabolism in mixotrophy. Therefore, CO_2_ fixation was skipped, as reflected by a significant down-regulation of gene expression. And these results experimentally verified the above assumption is valid in *C. zofingiensis* and provide a reference for research in plants [47]. Many works have been done on directly engineering of RuBisCO to accelerate CO_2_ fixation rate [49, 50]. It was previously reported that under current atmospheric conditions, nearly 30% of the carbohydrates formed by C_3_ photosynthesis are lost via photorespiration [33, 51]. However, photorespiration is indispensable for photosynthetic organisms, since this pathway participates in photoprotection [32], amino acid biosynthesis [52] and removal of toxic intermediate metabolites [53]. Hence, reducing rather than eliminating photorespiration has become an attractive avenue for improving photosynthetic efficiency [26, 33, 51, 54]. Recent work has shown that re-engineering photorespiratory pathways can significantly increase biomass production in higher plants [55]. The present study has been the first to show that skipping RubisCO could significantly reduce NPQ and photorespiration, and provided a strong evidence that increase of light energy fixation can be achieved not only by directly increasing CO_2_ fixation or by modifying photorespiration [55], but also by skipping the photosynthesis rate-limiting steps. Collectively, this study not only elaborated the mixotrophic metabolic mechanisms of *C. zofingiensis*, but also provides a theoretical basis and new ideas for future research on photosynthesis and glucose metabolism, and provides a foundation for future industrial applications of mixotrophy.

## Conclusions

Under mixotrophic cultivation, photosynthesis and glucose metabolism occur in a dynamic balance, such that enhancement of one results in lowering of the other. Compared with photoautotrophy, intermediates of glycolysis were supposed directly enter the chloroplast and substitute for inorganic carbon fixed by RuBisCO for organic carbon metabolism in the chloroplast. Therefore, the main rate-limiting enzyme, RuBisCO, was skipped, which resulted in decreased energy dissipation via non-photochemical quenching. Finally, compared with heterotrophy, energy provided by photosynthesis reduced the need for TCA-derived ATP, so the metabolism of glucose was reduced. Collectively, our results reveal a novel mechanism underlying mixotrophic metabolism in *C. zofingiensis*.

## Materials and methods

### Strains and culture conditions

*Chromochloris zofingiensis* ATCC30412 was used in the present study, which was heterotrophically cultured in Kuhl medium with 5 g L^−1^ glucose for 4 days, and then maintained in the dark at 16 °C to serve as seed stock [56]. For seed activation, 5 mL stock were inoculated in 50 mL fresh Kuhl medium (pH 6.1) with 5 g L^−1^ glucose and cultured in the dark at 25 °C with 150 rpm orbital shaking for 4 days.

The seed cells were collected, washed, and re-suspended at a cell density of 0.6 g L^−1^. To identify the interactions between photosynthesis and glucose metabolism under mixotrophic cultivation, six culture conditions, including photoautotrophy (P), photoautotrophy with CO_2_ supplementation (P + CO_2_), heterotrophy (H), mixotrophy (M), mixotrophy with CO_2_ supplementation (M + CO_2_) and mixotrophy with 10 μM DCMU (M + DCUM), were used. The light intensity in photoautotrophy and mixotrophy was 100 μmol m^−2^ s^−1^ light and the CO_2_ supplementation was 1.5% CO_2_ mixed in air. To determine the sources of carbon in cells under different culture conditions, five culture conditions, including heterotrophy with U-13C glucose (H + C13Glu), mixotrophy with U-13C glucose (M + C13Glu), mixotrophy with U-13C glucose and CO_2_ supplementation (M + C13Glu + CO_2_), mixotrophy with 10 mmol L^−1 13^C-labeled NaHCO_3_ (M + C13NaHCO_3_ + Glu) and photoautotrophy with 10 mmol L^−1 13^C-labeled NaHCO_3_ (P + C13NaHCO_3_), were used.

### Determination of dry weight, glucose consumption, specific growth rate, biomass yield on glucose and energy fixation

3 mL of the culture at each time point was collected, and centrifuged at 5000×*g*, 2 min. The glucose concentration of was determined by DNS method [57]. The pelleted cells were collected, washed and then dried at 80 °C for 4 h before determining DW.

The specific growth rate was calculated according to Eq. (1).

The energy fixation was calculated according to Eq. (2), which was adopted from Zhang et al. [9], and defined as the total energy in the increased DW. The carbon content in Eq. (2) was measured by an element analyzer (Flash 2000 series, Thermo Scientific). And conversion factor was adopted from Seo et al. [58], as 47.7 kJ per 1 g organic carbon.

The biomass yield on glucose was calculated according to Eq. (3), and defined as the ratio of DW increase to glucose [11].

1 $${\text{Specific}}\;{\text{growth}}\;{\text{rate}}\left( {{\text{h}}^{ - 1} } \right) = {{\left( {\ln {\text{DW}}_{2} {-}\ln {\text{DW}}_{1} } \right)} \mathord{\left/ {\vphantom {{\left( {\ln {\text{DW}}_{2} {-}\ln {\text{DW}}_{1} } \right)} {\left( {t_{2} {-} \, t_{1} } \right)}}} \right. \kern-\nulldelimiterspace} {\left( {t_{2} {-} \, t_{1} } \right)}},$$

where DW_2_ and DW_1_ are DW (g L^−1^) at where DW_2_ and DW_1_ are DW (g L^−1^) at time of *t*_2_ and *t*_1_, respectively

2 $${\text{Energy}}\;{\text{fixation}}\;({\text{KJ}}\,{\text{L}}^{ - 1} ) = {\text{carbon}}\;{\text{content}} \times \left( {{\text{maximum}}\;{\text{DW}}\left( {{\text{g}}\,{\text{L}}^{ - 1} } \right) - {\text{initial}}\;{\text{DW}}\left( {{\text{g}}\,{\text{L}}^{ - 1} } \right)} \right) \times {\text{conversion}}\;{\text{ factor}}\left( {{\text{KJ}}\,{\text{g}}^{ - 1} } \right),$$

3 $${\text{Biomass}}\;{\text{yield}}\;{\text{on}}\;{\text{glucose}}\left( {{\text{g}}\,{\text{g}}^{ - 1} } \right) = {{\left( {{\text{maximum}}\;{\text{DW}}\left( {{\text{g}}\,{\text{L}}^{ - 1} } \right){-}{\text{initial}}\;{\text{DW}}\left( {{\text{g}}\,{\text{L}}^{ - 1} } \right)} \right)} \mathord{\left/ {\vphantom {{\left( {{\text{maximum}}\;{\text{DW}}\left( {{\text{g}}\,{\text{L}}^{ - 1} } \right){-}{\text{initial}}\;{\text{DW}}\left( {{\text{g}}\,{\text{L}}^{ - 1} } \right)} \right)} {{\text{glucose}}\;{\text{concentration}}\left( {{\text{g}}\,{\text{L}}^{ - 1} } \right)}}} \right. \kern-\nulldelimiterspace} {{\text{glucose}}\;{\text{concentration}}\left( {{\text{g}}\,{\text{L}}^{ - 1} } \right)}};$$

### RuBisCO activity

The measurement of RuBisCO activity was conducted according to Zhang et al. [9]. Briefly, of different samples was determined using a commercial chemical assay kit (Jiangsu Keming Biotechnology Institute, Suzhou, China). Cell pellets in 10 mL culture were collected and ground under liquid nitrogen. Then the broken cells were extracted by extraction buffer (1 mM EDTA, 20 mM KCl, 10 mM Tris–HCl, pH 7.8). Then centrifuged for 5 min (4 °C, 12,000×*g*), the supernatant was used for enzyme activity measurement.

RuBisCO activity was measured using a RuBisCO assay kit (Keming Biotechnology Institute, Suzhou, China), which was followed the Racker’s method [59].

### C13-labeled metabolite analysis

The samples (40 ± 1 mg) were homogenized in 10 volumes (vol/wt) of methanol/acetonitrile/water (5:3:2, v/v/v) solution with TissueLyser JX-24 (Jinxin, Shanghai, China). The homogenates were vortexed for 1 min, and then centrifuged at for 15 min (14,000×*g*, 4 °C). Supernatant was performed in UPLC-MS/MS analysis.

Waters Acquity UPLC (Waters, Milford, MA) equipped with a Waters Xevo-TQXS system and an Amide column (100 mm × 2.1 mm, 1.7 μm) was used in the present study. Two mobile-phases were used: (A) 10 mM ammonium acetate and 0.1% ammonium hydroxide in water; (B) 10 mM ammonium acetate and 0.1% ammonium hydroxide in acetonitrile/water (90/10, v/v). The elution gradient was 2 min, 100% B; 8 min, 0% B; 13 min, 0% B; 13.5 min, 100% B; 17 min, 100% B. The column temperature was 30 °C. The injection volume was 5 μL. The flow rate was maintained at 300 μL min^−1^.

The analysis eluted from column were ionized in an electro spray ionization source in negative mode (ESI−). The parameters of mass spectrometry are as follows, capillary voltage − 0.5 kV, cone gas flow 150 L/h, desolvation temperature 500 °C, source temperature 150 °C, collision gas flow 0.15 mL/min, desolvation gas flow 600 L/h, and nebulizer gas flow 7 bar. The dwelling time was set at 0.025 s. Micromass Masslynx (version 4.2) was used to control instruments and acquire data.

Micromass Masslynx v4.2 was used to analyze data. Using the default parameters and assisting manual inspection to ensure the qualitative and quantitative accuracy of each compound, extract and output chromatographic retention time and peak area. The C13 labeled ratio can be calculated by the peak area of the same metabolite with different C13 labeled number. For instance, the peak areas of unlabeled, one carbon labeled, two carbon labeled, three carbon labeled and four carbon labeled E4P were *a*, *b*, *c*, *d* and e, respectively, then the C13 labeled ratio of E4P = (0*a* + 1*b* + 2*c* + 3*d* + 4*e*)/(4 × (*a* + *b* + *c* + *d* + *e*)).

### Chlorophyll fluorescence parameters

The chlorophyll fluorescence parameters including Y(II), Y(NPQ) and Y(NO) were measured using a PAM (pulse amplitude modulated fluorometry, PAM-2500, Heinz Walz GmbH, 91090, Effeltrich, German) [60]. Briefly, the cells of each sample were collected and re-suspended to about 1 absorbance at 680 nm, and then dark-adapted for 20 min, Minimal fluorescence yield (*F*_0_) was determined. The saturating light pulse was applied to the dark-adapted samples to obtain the maximal fluorescence yield (*F*_m_). Then actinic light was provided by the PAM 2500 with the intensity of 100 μmol m^−2^ s^−1^, and a saturation pulse was conducted every 30 s over a time span of 5 min, the maximal fluorescence was recorded as *F*_m_’ and the steady fluorescence was recorded as *F*_s_. Then Y(II), Y(NPQ) and Y(NO) were calculated by the following equations: ${\text{Y}}\left( {{\text{II}}} \right)\, = \,(F^{\prime}_{{\text{m}}} \, - \,F_{{\text{s}}} )/F^{\prime}_{{\text{m}}} ,$ ${\text{Y}}\left( {{\text{NPQ}}} \right)\, = \,F/F^{\prime}_{{\text{m}}} \, - \,F_{{\text{s}}} /F_{{\text{m}}} ,$ Y(NO) = *F*_s_/*F*_m_.

### RNA isolation and quality control

5 mL of culture collected at each time point was centrifuged for 3 min (5000×*g*, 4 °C), after which the supernatant was discarded. Then, the pelleted cells were rapidly frozen and ground with liquid nitrogen protection, and transferred to an RNase-free centrifuge tube. RNA was extracted using MiniBEST Plant RNA Extraction Kit (TaKaRa). The concentration and purity of RNA were determined using a NanoDrop 2000, while RNA integrity was assessed by agarose gel electrophoresis. The RIN value was measured using an Agilent 2100 Bioanalyzer. The distinct bands of RNA samples (free of impurities, RIN values > 9.0) were diluted to concentrations ranging from 300–1300 ng μL^−1^, and used for RNA library construction. Purified RNA was stored at − 80 °C prior to sequencing.

### cDNA library construction

5 μg of RNA was taken from each sample, and an RNA library was constructed using an Illumina TruSeqTM RNA sample preparation kit. The messenger RNA (mRNA) was enriched and randomly broke into small fragments (about 300 bp). Subsequently, an Invitrogen SuperScript double-stranded cDNA synthesis kit and Illumina random primers were used to synthesize double-stranded cDNA. The target library was PCR amplified using Phusion DNA polymerase (NEB), after which PCR products were separated by electrophoresis on 2% agarose gels. Target bands were gel-extracted and the recovered cDNA libraries were quantified by TBS380 (Picogreen) and subsequently sequenced using an Illumina HiSeq 4000.

### Read mapping, differential expression analysis and functional enrichment

The clean reads were achieved using SeqPrep and Sickle with default parameters. Then separately aligned to the *C. zofingiensis* reference genome using TopHat version 2.0.0 software [61]. (The genome sequence was supplied as Additional file 5 and all unigene sequences were supplied as Additional file 6).

To identify differentially expressed genes (DEGs), the expression level was defined using FRKM (fragments per kilobase of exon per million mapped reads) method. RSEM [62] was used to quantify the gene abundance. EdgeR (Empirical analysis of Digital Gene Expression in R) [63] was used to preform the differential expression analysis. In addition, the functional enrichment using KEGG (Kyoto Encyclopedia of Genes and Genomes) was conducted to identify the significantly enriched DEGs in metabolic pathways [64].

### Statistical analysis

All experiments were conducted with three biological replicates. Figure results reflect mean values ± SD. The statistical significance of the results was tested by *t*-test (*p* < 0.05) using SPSS version 19.0 (SPSS Inc., Chicago, USA).

## Supplementary Information

**Additional file 1: Table S1.** Changes of gene expression in photorespiration pathway under mixotrophic cultivation compared with photoautotrophic cultivation.

**Additional file 2: Figure S1.** Sample correlation of transcriptomes among the three trophic modes.

**Additional file 3: Figure S2.** KEGG enrichment of different expressed genes in M vs. P and M vs. H.

**Additional file 4.** The trinity ID, FPKM and log_2_FC^a^ of genes involved in Fig. 5.

**Additional file 5.** The genome sequence of *C. zofingiensis* ATCC 30412.

**Additional file 6.** Unigene sequences of *C. zofingiensis* ATCC 30412.

## Data Availability

All data supporting the findings of this study are available within the paper and within its additional files published online.
