# Supplementary material for: Investigation of carbon and energy metabolic mechanism of mixotrophy in Chromochloris zofingiensis
Source: Biotechnol Biofuels. 2021 Feb 4;14:36. doi: 10.1186/s13068-021-01890-5 (PMC7863362; doi:10.1186/s13068-021-01890-5)
Supplement: Supplementary file 1 — Additional file 1: Table S1. Changes of gene expression in photorespiration pathway under mixotrophic cultivation compared with photoautotrophic cultivation. [file 13068_2021_1890_MOESM1_ESM.docx]

**Table S1:** Changes of gene expression in photorespiration pathway under mixotrophic cultivation compared with photoautotrophic cultivation (For genes with multiple copies, only the one with the highest expression is listed).

| Compartment | Gene name | Trinity ID | Log_2_FC^a^ |
| --- | --- | --- | --- |
| Chloroplast | RuBisCO subunit | Cz.scaffold68.128 | -0.61 |
|  | RuBisCO activase | Cz.scaffold25.371 | -1.98 |
|  | Phosphoglycolate phosphatase | Cz.scaffold64.444 | -0.94 |
|  | Glycerate 3-kinase | Cz.scaffold6.97 | -5.76 |
| Peroxisome | Glycolate oxidase | Cz.scaffold64.388 | -0.33 |
|  | Glutamate:glyoxylate aminotransferase | Cz.scaffold64.996 | -2.97 |
|  | Serine:glyoxylate aminotransferase | Cz.scaffold34.255 | -1.67 |
|  | Peroxisomal hydroxypyruvate reductase | Cz.scaffold8.133 | -1.74 |
| Mitochondrion | Serine hydroxymethyltransferase | Cz.scaffold58.435 | -2.43 |
|  | Glycine decarboxylase | Cz.scaffold55.128 | -2.75 |
