# Supplementary material for: Investigation of carbon and energy metabolic mechanism of mixotrophy in Chromochloris zofingiensis
Source: Biotechnol Biofuels. 2021 Feb 4;14:36. doi: 10.1186/s13068-021-01890-5 (PMC7863362; doi:10.1186/s13068-021-01890-5)
Supplement: Supplementary file 2 — Additional file 2: Figure S1. Sample correlation of transcriptomes among the three trophic modes. [file 13068_2021_1890_MOESM2_ESM.docx]

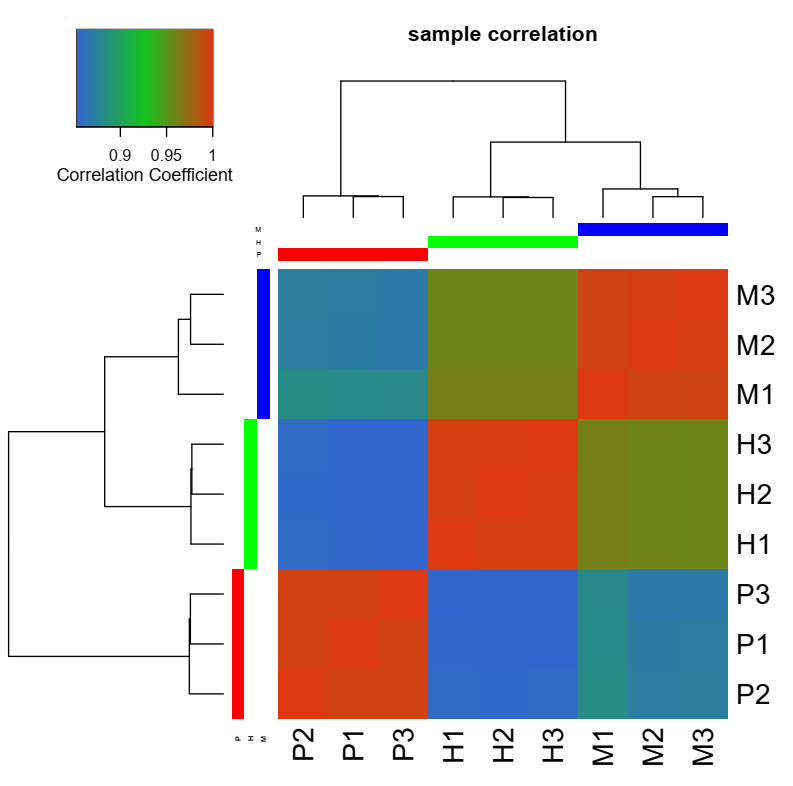


**Figure S1:** Sample correlation of transcriptomes among the three trophic modes (P: photoautotrophogy; H: heterotrophy; M: mixotrophy. The numbers after the symbols represent different biological replicates).
