# Supplementary material for: Investigation of carbon and energy metabolic mechanism of mixotrophy in Chromochloris zofingiensis
Source: Biotechnol Biofuels. 2021 Feb 4;14:36. doi: 10.1186/s13068-021-01890-5 (PMC7863362; doi:10.1186/s13068-021-01890-5)
Supplement: Supplementary file 3 — Additional file 3: Figure S2. KEGG enrichment of different expressed genes in M vs. P and M vs. H. [file 13068_2021_1890_MOESM3_ESM.docx]

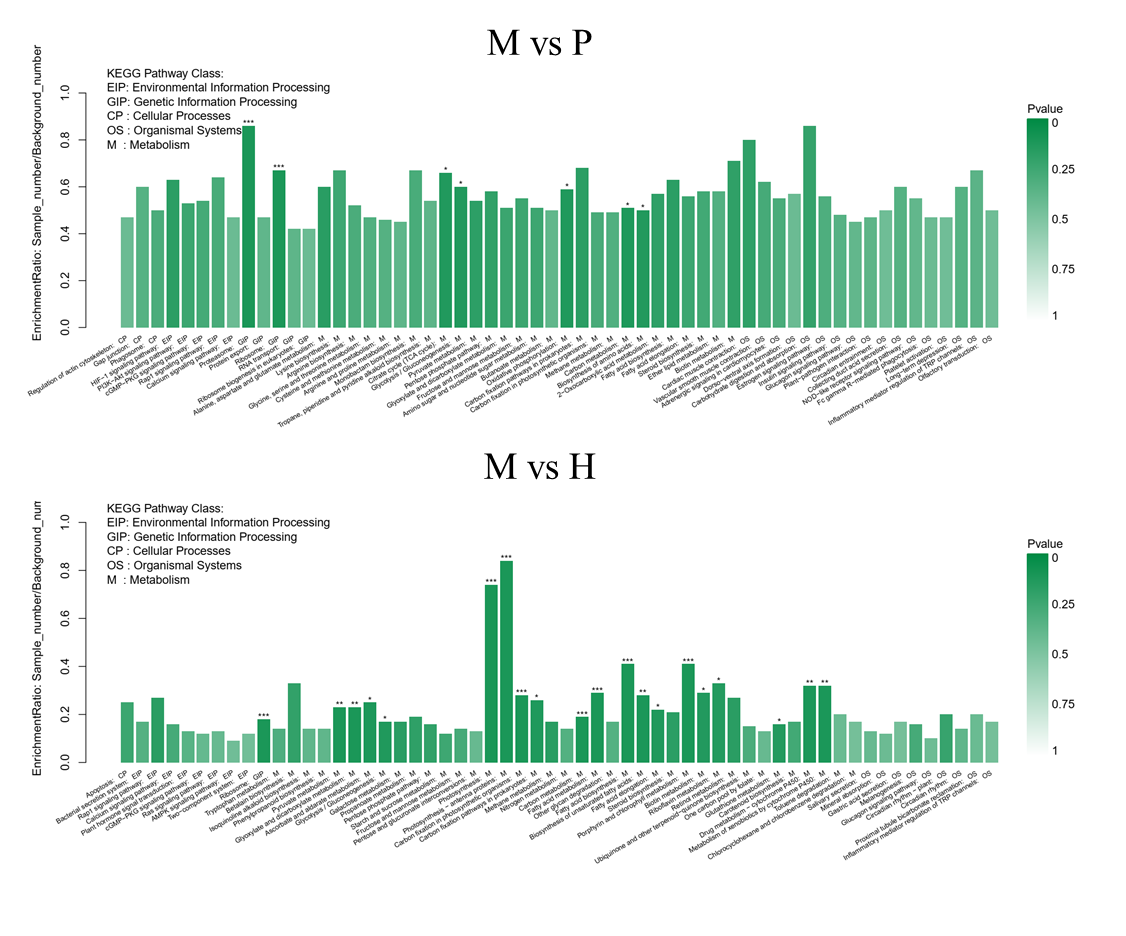


**Figure S2:** KEGG enrichment of different expressed genes in M vs P and M vs H (*** significantly enriched P<0.001; ** significantly enriched P<0.01; * significantly enriched P<0.05).
